# Selective versus routine use of episiotomy for vaginal births in Shanghai hospitals, China: a comparison of policies

**DOI:** 10.1186/s12884-022-04790-0

**Published:** 2022-06-11

**Authors:** Shengyi Gu, Jindan Pei, Chenchen Zhou, Xiaobo Zhao, Sheng Wan, Jun Zhang, Adewumi Adanlawo, Zhongcheng Luo, Guizhu Wu, Xiaolin Hua

**Affiliations:** 1 Department of Obstetrics and Gynecology, Shanghai First Maternity and Infant Hospital, School of Medicine, Tongji University, Shanghai, 200092 China; 2 Ministry of Education-Shanghai Key Laboratory of Children’s Environmental Health, and Department of Obstetrics, Xinhua Hospital, Shanghai Jiao-Tong University School of Medicine, Shanghai, 200092 China; 3 Department of Obstetrics and Gynecology, Saskatchewan Health Authority, Regina, SK Canada; 4 Lunenfeld-Tanenbaum Research Institute, Prosserman Centre for Population Health Research, Department of Obstetrics and Gynecology, Mount Sinai Hospital, Faculty of Medicine, University of Toronto, Toronto, M5G 1X5 Canada

**Keywords:** Vaginal delivery, Selective episiotomy, Routine episiotomy, Perineal lacerations, Cohort study

## Abstract

**Background:**

To assess the effects of policy of selective versus routine episiotomy on mother and baby for women delivering vaginally in Shanghai and whether the hospital type has any effect on the outcomes.

**Method:**

This was a multi-center retrospective cohort study in Shanghai between March 2015 and May 2017. The study population were vaginal births with selective or routine episiotomy (*n* = 5478) in 20 secondary or tertiary hospitals. Main Outcome Measure was the incidence of severe perineal lacerations. The adjusted odds ratios (aOR) and 95% confidence intervals (CI) were estimated by logistic regression and presented as the effect sizes. All models were stratified by the utilization of level (secondary and tertiary) and type (general and Obstetric) of hospital.

**Results:**

The primary outcome was not significantly different between vaginal births with routine and selective episiotomy. Patients with selective episiotomy had a lower risk of postpartum hemorrhage, and newborns in the selective episiotomy group had a lower risk of shoulder dystocia and Neonatal Ward compared to those with routine episiotomy. Newborns in selective episiotomy group had a lower risk of birth injury in tertiary hospital. However, newborns in selective episiotomy group had a higher risk of birth injury in general hospitals.

**Conclusion:**

Selective episiotomy is safe and can be recommended over routine episiotomy in obstetric and tertiary hospital settings in China.

**Supplementary Information:**

The online version contains supplementary material available at 10.1186/s12884-022-04790-0.

## Introduction

Vaginal delivery is considered a preferred method of delivery, although cesarean delivery has been increasing over the recent decades. Compared to cesarean delivery, which may be necessary in order to prevent adverse outcomes or to save life, the primary maternal benefit of vaginal delivery is less anesthetic and surgical complications, including less hemorrhage requiring transfusion or hysterectomy, less bowel or bladder injury, less postoperative ileus, less amniotic fluid embolism, less air embolism and less thromboembolic disease [1, 2]. Other maternal benefit of vaginal delivery is less long-term risks including less uterine rupture, less recurrent cesarean delivery, lower abnormal placentation risks including previa/accreta [3, 4]. Fetal benefits include higher likelihood of breast-feeding, shorter hospital admission, decreased rates of respiratory distress syndrome, and lower risk of respiratory-related NICU admission [2]. While several problems have been reported with vaginal birth. A major problem in vaginal birth is the risk of vagina and perineal tears leading to pain, bleeding, infection, dyspareunia and even a prolonged hospital stay. Subsequently, severe perineal tears increase the risk of prolapse of pelvic organs and fecal incontinence [5].

Episiotomy is a surgical incision performed during the delivery process to expand the vaginal opening in order to reduce the risk of lacerations of the posterior wall and severe perineal lacerations (3rd or 4th degree) to avoid injury to the pelvic floor and facilitate the birth of the baby [6, 7]. It can provide enough space to allow for assisted deliveries with forceps or vacuums to shorten labor and reduce pain [8, 9]. However, with routine use of episiotomy, the rate of unnecessary perineal tear is increased. Studies have shown that the routine use of episiotomy is associated with a higher prevalence of posterior wall perineal tear and severe perineal tear [10–12]. The potential long-term complications of episiotomy include dyspareunia, anorectal dysfunction and sexual dysfunction [13]. Therefore, routine episiotomy rates in Western developed countries have dropped from 70 to 15% since 1983 [14].

To better refine the use of episiotomy, selective use of episiotomy was advocated. Several studies have shown that selective use of episiotomy was preferable to routine episiotomy [15] with respect to the risk of lacerations of the posterior wall and unnecessary perineal lacerations [16]. However, in places where marked reduction in the rate of episiotomy was achieved, there was an increase in the occurrence of severe perineal lacerations [17]. Ethnicity may play a role in the long term effects of perineal injury as described by Abdool [18]. The perineal tear is closely related not only to the size and the position of the fetus, and the size of the pelvis of the woman, but also to the conditions of the perineal-associated muscles and ligaments. Whether selective use of episiotomy is better than routine use of episiotomy has not been systematically assessed in different ethnic groups worldwide.

In China, the rate cesarean increased from 28.8% in 2008 to 34.9% in 2014 [19], a significant proportion of which were without medical indication. However, recent studies have shown that vaginal births have become more frequent in recent since the promulgation of the two-child policy in 2015 [20]. Nowadays, selective episiotomy is preferred over routine episiotomy in most developed countries, However, it is uncertain whether selective episiotomy should be promoted in China considering the differences between ethnic groups concerning the risk of perineal laceration following vaginal delivery [21]. The aim of the present study was to assess the effects of policy of selective versus routine episiotomy on mother and baby for women delivering vaginally in Shanghai and whether the hospital type has any effect on the outcomes.

## Methods

### Study population

The was a multi-center, retrospective cohort study in Shanghai between March 2015 and May 2017. The participating hospitals were solicited through obstetric conferences and networks. Those that expressed an interest were asked to provide basic information about the hospitals. There was a total of 20 participating hospitals, including 13 secondary and 7 tertiary hospitals (11 general hospitals and 9 obstetric hospitals). Eligible subjects were singleton pregnancies at 34^+^ weeks of gestation in primiparous women with vaginal deliveries in cephalic presentation. Medical records were retrieved and reviewed, and information was extracted by trained research nurses. Exclusion criteria included multiple pregnancy, non-cephalic pregnancy, gestation weeks > 42, age < 18 or age > 45.This methodology has been used in the WHO Global Survey of Maternal and Perinatal Health and the WHO Multi-Country Survey of Maternal and Newborn Health [22, 23]. A data coordination center was responsible for the management and maintenance of the database and website, coordination among hospitals, investigators’ training, and quality control of the data. A logic check function was programmed in the database for preventing missing items and data entry errors. Data administrators checked the data regularly and contacted the hospital investigators to fill in omissions or correct errors.

### Covariates

Individual covariates were maternal characteristics including maternal age, body mass index (BMI), pre-existing medical conditions such as diabetes, cardiac diseases, hyperthyroidism or hypothyroidism, birthweight, thyroid disease, and gestational complications. Institute covariates were the level (secondary vs. tertiary) and type of hospital (general vs. obstetric).

### Exposures and outcomes

The exposure variable was the hospital policy for selective vs. routine episiotomy. Selective episiotomy mean episiotomy was done when the obstetrician deems it necessary. Indications of selective episiotomy include the size of fetus, perineal conditions, and the possibility of vaginal assistance. Routine episiotomy mean that all women had an episiotomy. The group was determined by hospital policy. There were 10 hospitals in the group of routine episiotomy and 10 in the group of selective episiotomy.

The primary outcome was severe perineal laceration (3rd or 4th degree) and/or vaginal trauma [24]. Secondary outcomes were major adverse maternal and neonatal outcomes, including meconium-stained amniotic fluid, fetal distress, shoulder dystocia, dehiscence, postpartum hemorrhage, transfusion, puerperal infection, amniotic fluid embolism, maternal thrombosis, admission into intensive care unit, birth injury, 5 min APGAR score ≤ 7, and admission to NICU.

### Statistical analysis

To better represent selective episiotomy with routine episiotomy in Shanghai overall, we calculated a weight for each woman in the survey using the number of deliveries in each hospital. Unweighted results were showed in supplementary material.

Hospital level and type was considered as a post stratification factor. Adverse maternal and infant outcomes in routine episiotomy vs. selective episiotomy were compared by chi-square tests. Multivariable logistic regression was used to estimate the effects of selective vs. routine episiotomy on adverse maternal and infant outcomes, adjusting for potential confounders. Potential confounders included maternal age at childbirth, BMI, use of assisted reproductive technology, induced labor, pregnancy-induced hypertension, preexisting diabetes, gestational diabetes and pre-existing cardiac diseases. All analyses were performed using SPSS version 22.0 (IBM, Somers, NY). Two-tailed *P* values < 0.05 were considered statistically significant.

### Funding

This study was funded by the Shanghai Municipal Commission of Health and Family Planning (GWIV-26) and Pudong Commission of Health and Family Planning (PW2019D-13).

## Results

**Fig. 1:**
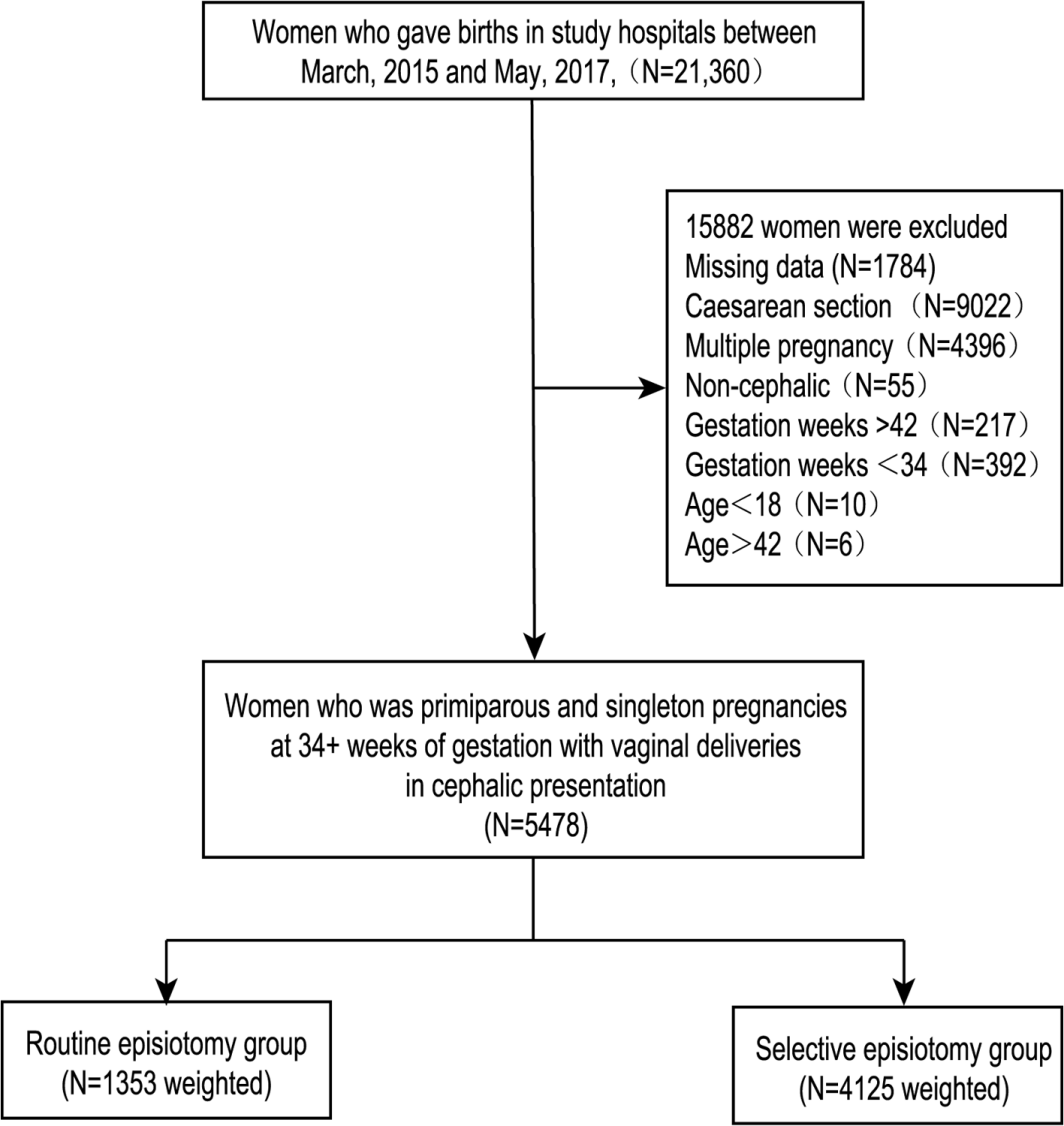
Study flow chart

A total of 21,360 women gave births in study hospitals between March, 2015 and May, 2017, and 5478 women met the study inclusion criteria and exclusion criteria. Of these, 75.3% patients were assessed with policy of selective episiotomy, 24.7% patients were treated with policy of routine episiotomy (Fig. 1).

Demographic and clinical characteristics of study participants are shown in Table 1 and Table S1. The patients with selective episiotomy had advanced age compared to the patients with routine episiotomy (27.10 vs 27.94, *P <* 0.05). Pre-existing cardiac diseases was more common in women with selective episiotomy (0.55% vs 1.09%, *P <* 0.05).

**Table 1:** Characteristics at baseline (weighted)

Characteristics	Routine episiotomy (1353)		Selective episiotomy (4125)		P
	mean	95%CI	mean	95%CI	
age	27.96	27.82–28.11	28.32	28.22–28.43	< 0.05
-20	33	2.44	34	0.83	
20–34	1283	94.86	4000	96.95	
35-	36	2.70	92	2.22	
BMI	21.22	21–21.34	21.18	21.09–21.27	0.60
−18.5	206	15	522	12.64	
18.5–24	936	69	3079	74.63	
24-	210	16	525	12.73	
Diabetes	4	0.32	6	0.15	0.21
	n	%	n	%	P
Pre-existing cardiac diseases	7	0.54	73	1.77	< 0.05
Pre-existing renal disease	3	0.21	10	0.25	0.84
Pre-existing autoimmune disease	2	0.16	6	0.15	0.90
Pre-existing hyperthyroidism	6	0.46	24	0.58	0.61
Pre-existing hypothyroidism	17	1.25	59	1.42	0.63

*BMI* Body mass index

The incidences of pregnancy complications are presented in Table 2 and Table S2. More women had hypothyroidism (8.7% vs 5.55%, *P <* 0.05) and pregnancy-induced hypertension syndrome (14.34% vs 12.14%, *P <* 0.05) in the routine episiotomy group.

**Table 2:** Current pregnancy complications (weighted)

Characteristics	Routine episiotomy (1353)		Selective episiotomy (4125)		P
	n	%	n	%	
ART	19	1.38	88	2.13	0.08
Hyperthyroidism	8	0.61	26	0.63	0.96
Hypothyroidism	99	7.29	202	4.89	0.05
Other thyroidism	14	1.03	68	1.65	0.10
PIH	191	14.1	496	12.02	< 0.05
GDM	146	10.82	415	10.05	0.42
placental abruption	1	0.05	9	0.22	0.19
PROM	317	23.42	1022	24.77	0.32
Prenatal stillbirth	0	0.05	3	0.08	0.39

*ART* Assisted reproductive technology, *PIH* Pregnancy-induced hypertension syndrome, *GDM* Gestational Diabetes Mellitus, *PROM* Premature rupture of membranes

The incidence rates of adverse maternal and neonatal outcomes are presented in Tables 3, 4 and Table S3. The rate of episiotomy in the selective episiotomy group was 33.55%, and those in routine episiotomy group was 81.84%. The incidence rate of severe perineal laceration was lower in the selective episiotomy, comvs routine episiotomypared to routine episiotomy. Besides, shoulder dystocia, postpartum hemorrhage and neonatal ward were less frequent in newborns and pregnant women in selective episiotomy group. And more women had doula and term labor in the selective episiotomy group.

**Table 3:** Selective versus Routine episiotomy: maternal (weighted)

Characteristics	Routine episiotomy (1353)		Selective episiotomy (4125)		P
	n	%	n	%	
Severe perineal laceration	3	0.23	2	0.04	< 0.05
Term labor	375	27.7	1462	35.43	< 0.05
Doula	127	9.4	2622	63.55	< 0.05
Meconium	96	7.09	283	6.87	0.78
Placenta accreta	24	1.8	88	2.14	0.44
Incompletely uterine rupture	1	0.08	7	0.17	0.44
Episiotomy	1107	81.84	1384	33.55	< 0.05
Postpartum hemorrhage	48	3.52	68	1.65	< 0.05
Transfusion	6	0.48	14	0.35	0.48
Puerperal infection	3	0.19	20	0.48	0.15
Vacuum or forceps delivery	66	4.90	184	4.48	0.22

**Table 4:** Selective versus Routine episiotomy: neonatal outcomes (weighted)

Characteristics	Routine episiotomy (1353)		Selective episiotomy (4125)		P
	mean	95%CI	mean	95%CI	
Birthweight	3277.6	3261.8–3293.4	3295.9	3283.1–3308.6	0.12
	n	%	n	%	
Fetal distress	80	5.94	190	4.6	0.05
Shoulder dystocia	13	0.97	4	0.1	< 0.05
Birthweight	3277.6	3261.8–3293.4	3295.9	3283.1–3308.6	0.12
Apgar≤7 (5 min)	2	0.12	11	0.28	0.29
Birth injury	243	17.96	708	17.15	0.49
Congenital malformation	30	2.19	65	1.58	0.13
Neonatal Ward	159	11.77	302	7.32	< 0.05

**Fig. 2:**
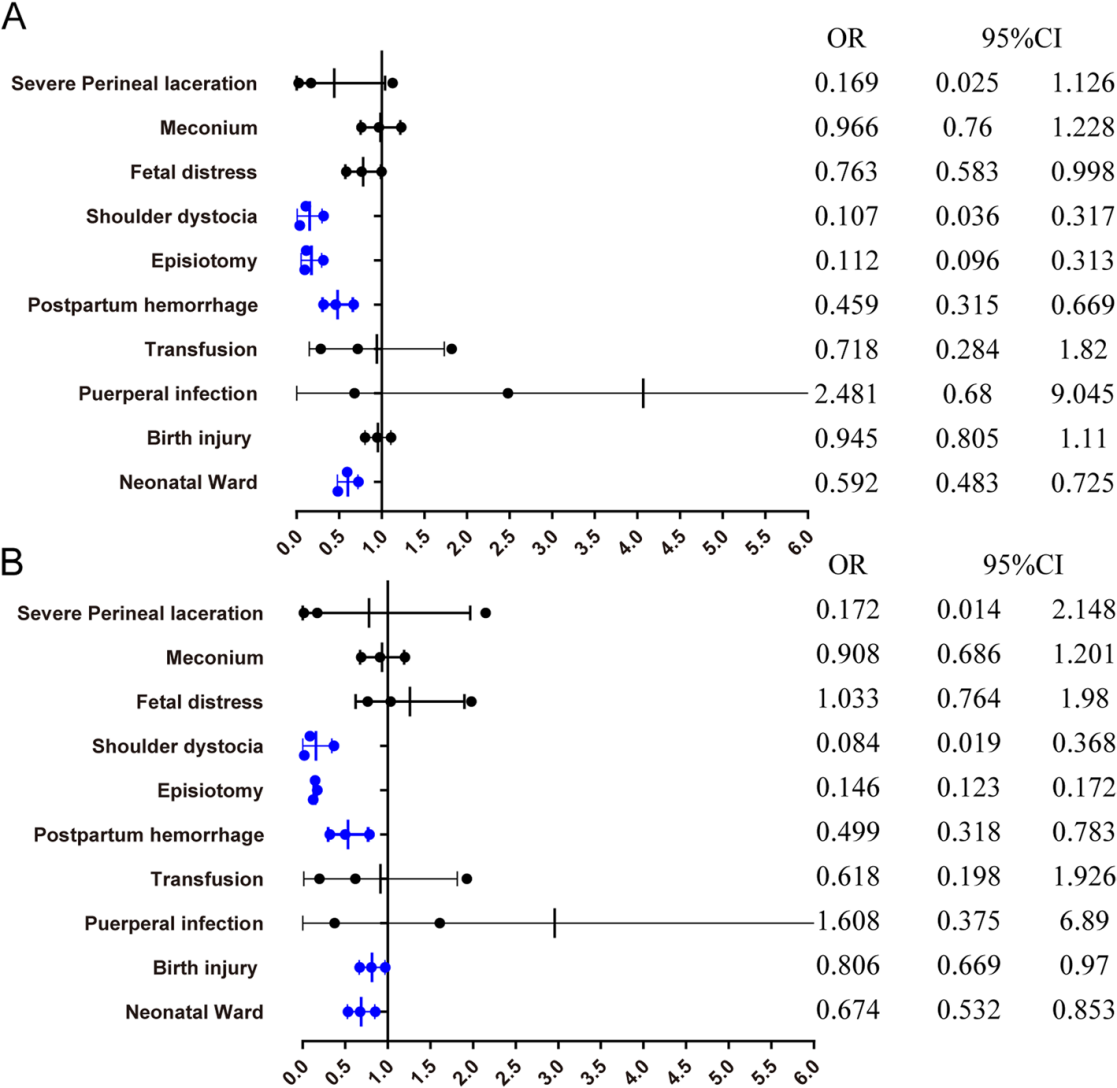
Selective versus Routine episiotomy: maternal and neonatal outcomes (weighted). **A.** Weighted data, crude ORs. **B**. Weighted data, adjusted ORs. The ORs were adjusted for maternal age at childbirth, BMI, the use of assisted reproductive technology, induced labor, pregnancy-induced hypertension, preexisting diabetes, hypothyroidism, pre-existing cardiac diseases and Placenta accrete

The outcomes comparing selective vs. routine episiotomy are showed in Fig. 2 and Table S4. The risk of severe perineal laceration was not significant different between selective vs. routine episiotomy, but the incidence rates were lower for shoulder dystocia (aOR = 0.084,95%CI:0.059–0.368), postpartum hemorrhage (aOR = 0.499,95%CI:0.318–0.783). And newborns in the selective episiotomy group are at lower risk of neonatal ward admission (aOR = 0.674,95%CI:0.532–0.853).

**Fig. 3:**
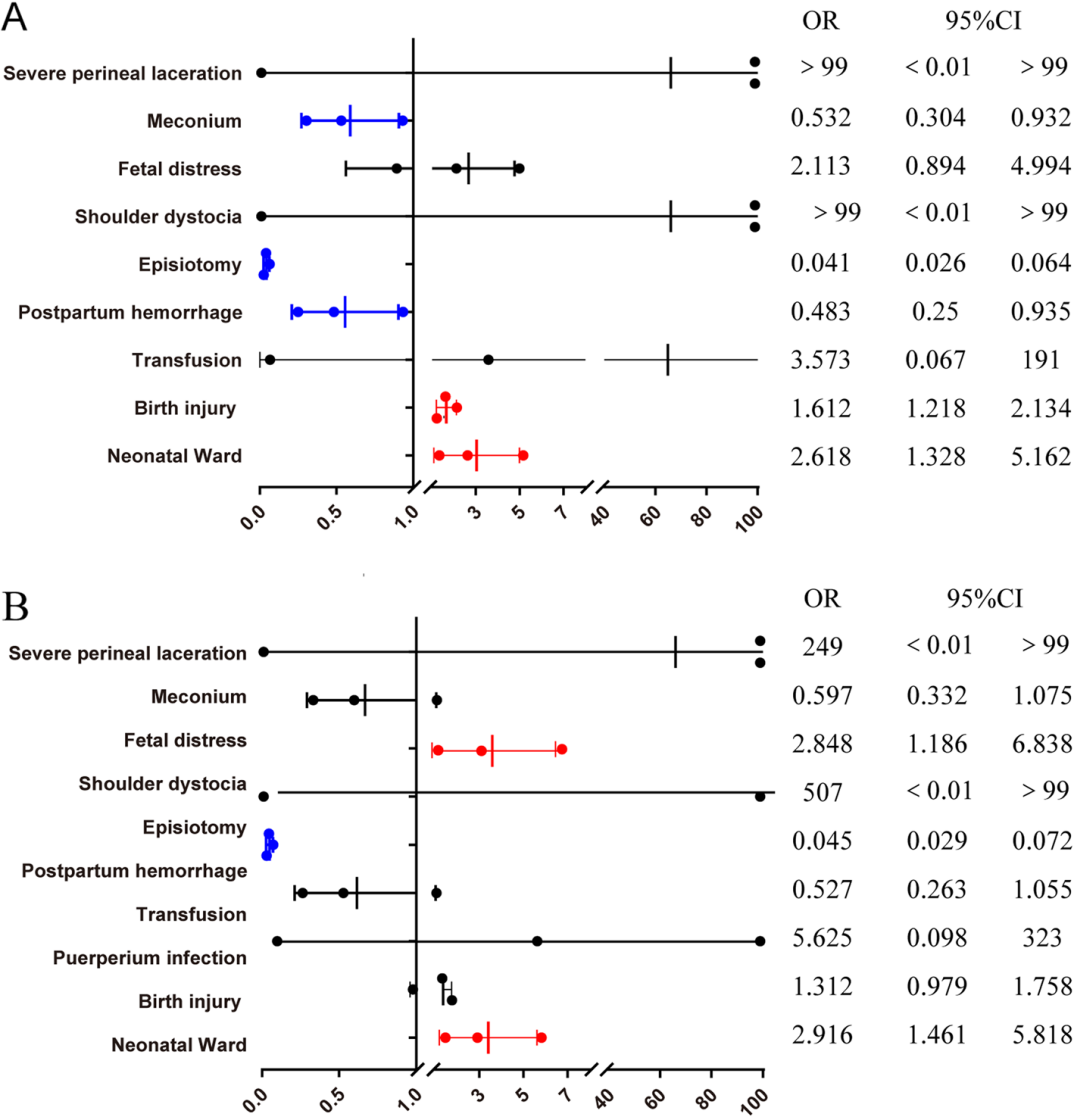
Selective versus Routine episiotomy in secondary hospitals: maternal and neonatal outcomes (weighted). **A.** Weighted data, crude ORs. **B.** Weighted data, adjusted ORs. The ORs were adjusted for maternal age at childbirth, BMI, the use of assisted reproductive technology, induced labor, pregnancy-induced hypertension, preexisting diabetes, hypothyroidism, pre-existing cardiac diseases and Placenta accrete

**Fig. 4:**
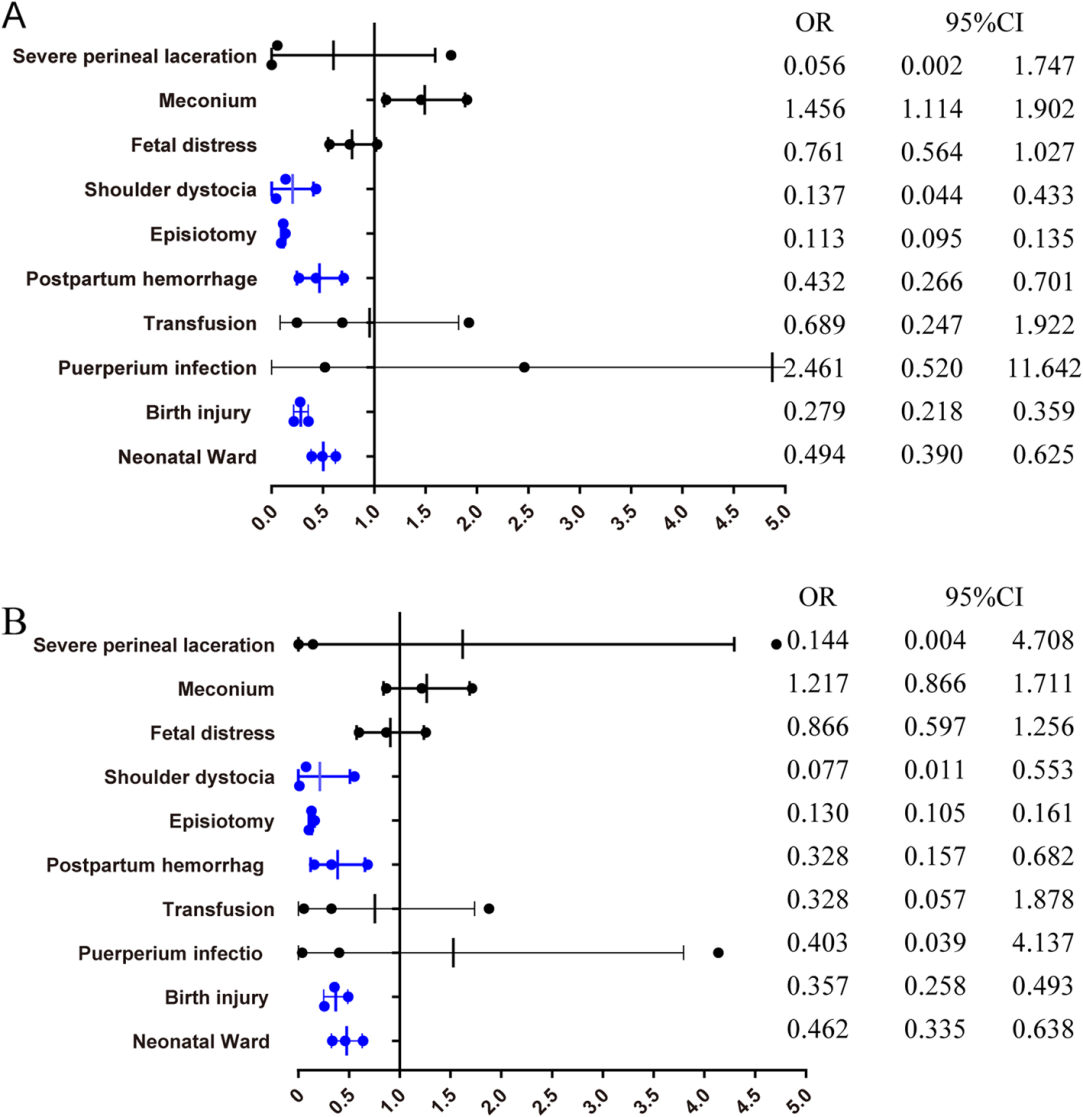
Selective versus Routine episiotomy in tertiary hospitals: maternal and neonatal outcomes (weighted). **A.** Weighted data, crude ORs. **B.** Weighted data, adjusted ORs. The ORs were adjusted for maternal age at childbirth, BMI, the use of assisted reproductive technology, induced labor, pregnancy-induced hypertension, preexisting diabetes, hypothyroidism, pre-existing cardiac diseases and Placenta accrete

Comparisons of maternal and neonatal outcomes between selective vs routine episiotomy in secondary and tertiary hospitals are presented in Tables 5, 6, Figs. 3, 4, Table S5, and Table S6. In secondary hospitals, the rate of episiotomy in the selective episiotomy group was 38.93%, and those in routine episiotomy group was 94.01%. While in tertiary hospitals, the rate of episiotomy in the selective episiotomy group was 28.24%, and those in routine episiotomy group was 77.69%. The risk of severe perineal laceration was not significant different between selective and routine episiotomy in both secondary and tertiary hospitals. In secondary hospitals, newborns in the selective episiotomy group were at higher risk of neonatal ward admission; but in tertiary hospitals, newborns in the selective episiotomy group were in lower risk of neonatal ward (aOR = 0.462,95%CI:0.335–0.638), birth injury (aOR = 0.357,95%CI:0.258–0.493), postpartum hemorrhage (aOR = 0.328,95%CI:0.157–0.682), and shoulder dystocia (aOR =0.077,95%CI:0.051–0.553).

**Table 5:** Selective versus Routine episiotomy in Secondary hospitals and Tertiary hospitals: maternal outcomes (weighted)

Characteristics	Secondary hospitals (2393)					Tertiary hospitals(3085)				
	Routine episiotomy(343)		Selective episiotomy (2050)		P	Routine episiotomy(1010)		Routine episiotomy(2075)		P
Severe perineal laceration	0	0	1	0.06	0.65	4	0.31	0	0	< 0.05
Term labor	83	24.25	630	30.72	< 0.05	291	28.87	832	40.09	< 0.05
Doula	104	30.28	1287	62.86	< 0.05	23	2.28	1334	64.27	< 0.05
Meconium	18	4.86	52	2.65	0.03	79	7.85	229	11.03	< 0.05
Placenta accreta	4	1.27	38	1.83	0.46	20	1.98	51	2.44	0.42
Incompletely uterine rupture	1	0.16	3	0.13	0.89	1	0.05	4	0.20	0.30
Episiotomy	323	94.01	798	38.93	< 0.05	784	77.69	586	28.24	< 0.05
Postpartum hemorrhage	12	3.55	36	1.75	< 0.05	35	3.50	32	1.54	< 0.05
Transfusion	1	0.07	5	0.26	0.50	6	0.62	9	0.43	0.47
Puerperal infection	1	0.20	11	0.49	0.46	2	0.19	10	0.46	0.24

**Table 6:** Selective versus Routine episiotomy in Secondary hospitals and Tertiary hospitals: neonatal outcomes (weighted)

Characteristics	Secondary hospitals (2393)					Tertiary hospitals(3085)				
	Routine episiotomy(343)		Selective episiotomy (2050)		P	Routine episiotomy(1010)		Routine episiotomy(2075)		P
	mean	95%CI	mean	95%CI		mean	95%CI	mean	95%CI	
Birthweight	3280.5	3257.9–3303.2	3295.6	3278.5–3312.8	0.46	3276.6	3255.6–3297.6	3296.1	3277.1–3315.0	0.20
	n	%	n	%		n	%	n	%	n
Shoulder dystocia	0	0	1	0.03	0.76	13	1.30	4	0.18	< 0.05
Apgar≤7 (5 min)	0	0	5	0.22	0.38	2	0.15	7	0.33	0.37
Birth injury	69	20.18	594	28.96	< 0.05	174	17.21	114	5.49	< 0.05
Congenital malformation	2	0.53	22	1.08	0.34	28	2.76	43	2.07	0.23
Neonatal Ward	9	2.67	137	6.69	< 0.05	150	14.88	165	7.94	< 0.05

**Fig. 5:**
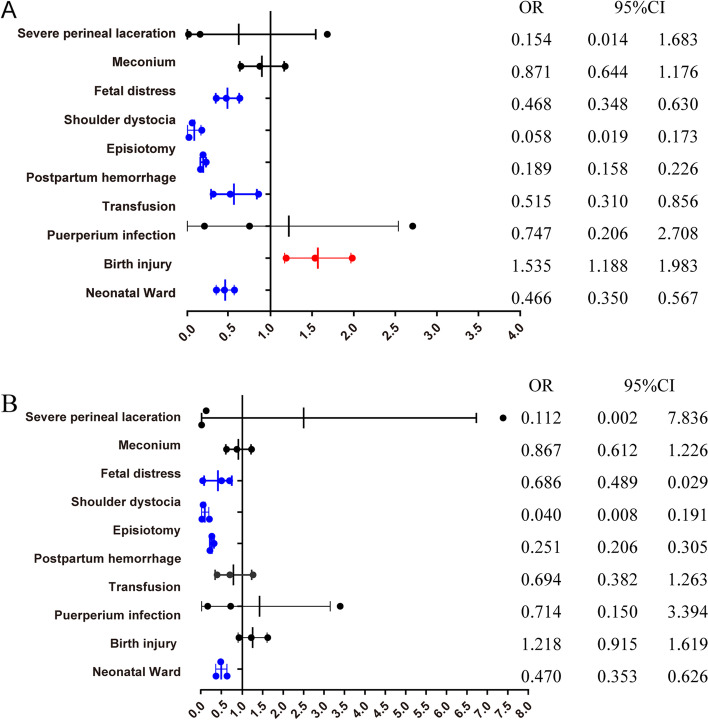
Selective versus Routine episiotomy in Obstetric hospitals: maternal and neonatal outcomes (weighted). **A.** Weighted data, crude ORs. **B.** Weighted data, adjusted ORs. The ORs were adjusted for maternal age at childbirth, BMI, the use of assisted reproductive technology, induced labor, pregnancy-induced hypertension, preexisting diabetes, hypothyroidism, pre-existing cardiac diseases and Placenta accrete

**Fig. 6:**
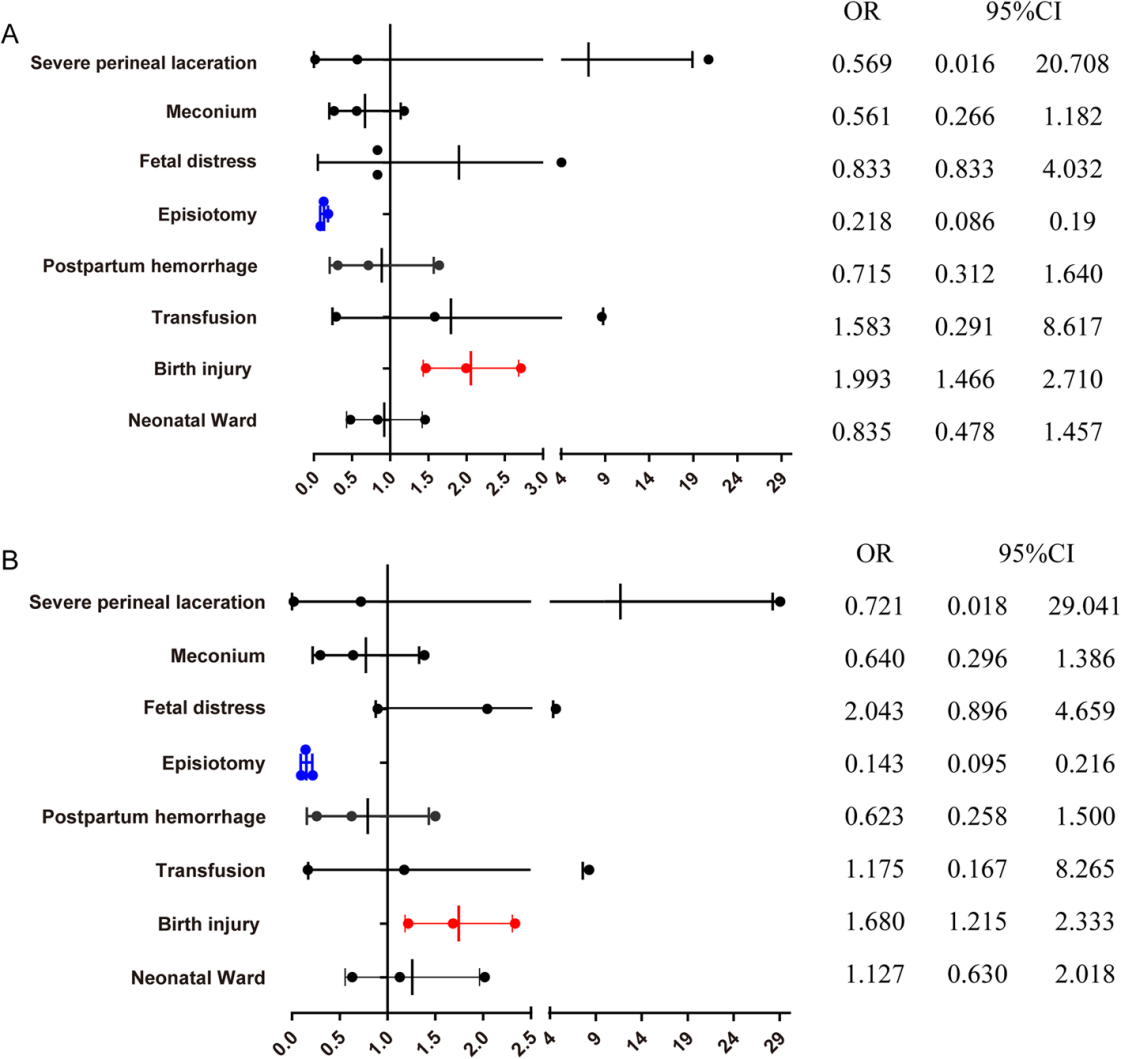
Selective versus Routine episiotomy in General hospitals: maternal and neonatal outcomes (weighted). **A.** Weighted data, crude ORs. **B.** Weighted data, adjusted ORs. The ORs were adjusted for maternal age at childbirth, BMI, the use of assisted reproductive technology, induced labor, pregnancy-induced hypertension, preexisting diabetes, hypothyroidism, pre-existing cardiac diseases and Placenta accrete

The incidence rates of maternal and neonatal outcomes comparing selective vs routine episiotomy in obstetric and general hospitals are presented in Tables 7, 8, Figs. 5, 6, Table S7, and Table S8. There was no significant difference in the primary outcome (severe perineal/vaginal trauma) between the two groups in both obstetric and general hospitals. In the obstetric hospitals, newborns in the selective episiotomy group had lower risks of neonatal ward (aOR = 0.47,95%CI:0.353–0.626), fetal distress (aOR = 0.686,95%CI:0.489–0.829), and shoulder dystocia (aOR = 0.04,95%CI:0.008–0.191), In contrast, newborns in the selective episiotomy group were in a higher risk of birth injury (aOR = 1.68,95%CI:1.215–2.333) in general hospitals.

**Table 7:** Selective versus Routine episiotomy in Obstetric hospitals and General hospitals: maternal outcomes (weighted)

Characteristics	Obstetric hospitals (4564)					General hospitals (914)				
	Routine episiotomy (689)		Selective episiotomy (3875)		P	Routine episiotomy (664)		Selective episiotomy (250)		P
	n	%	n	%		n	%	n	%	
Severe perineal laceration	1	0.21	1	0.03	0.08	2	0.25	1	0.14	0.76
Term labor	200	29.14	1361	35.12	< 0.05	174	26.21	101	40.37	< 0.05
Augment	229	33.27	841	21.70	< 0.05	94	14.12	65	26.2	< 0.05
Doula	40	5.89	2540	65.54	< 0.05	87	13.03	81	32.65	< 0.05
Meconium	55	8.05	275	7.08	0.37	41	6.09	9	3.51	0.12
Placenta accreta	14	2	83	2.15	0.80	11	1.58	5	1.91	0.74
Incompletely uterine rupture	0	0	7	0.18	0.27	1	0.15	0	0	0.54
Episiotomy	488	70.96	1226	31.64	< 0.05	619	93.09	158	63.27	< 0.05
Postpartum hemorrhage	21	2.99	61	1.56	< 0.05	27	4.06	7	2.94	0.43
Transfusion	3	0.42	12	0.31	0.66	4	0.55	2	0.86	0.59
Puerperal infection	2	0.29	19	0.49	0.47	1	0.10	1	0.29	0.50

**Table 8:** Selective versus Routine episiotomy in Obstetric hospitals and General hospitals: neonatal outcomes (weighted)

Characteristics	Obstetric hospitals (4564)					General hospitals (914)				
	Routine episiotomy (689)		Selective episiotomy (3875)		P	Routine episiotomy (664)		Selective episiotomy (250)		P
	mean	95%CI	mean	95%CI		mean	95%CI	mean	95%CI	
Birthweight	3281.0	3250.7–3311.3	3297.7	3283.5–3312.0	0.36	3274.1	3255.8–3292.3	3266.8	3238–3295.6	0.68
	n	%	n	%		n	%	n	%	n
Fetal distress	64	9.37	179	4.62	< 0.05	16	2.39	11	4.30	0.13
Shoulder dystocia	13	1.89	4	0.11	< 0.05	1	0.03	0	0	0.80
Apgar≤7 (5 min)	0	0	10	0.26	0.18	2	0.23	1	0.52	0.49
Birth injury	74	10.78	607	15.65	< 0.05	169	25.39	101	40.41	< 0.05
Congenital malformation	10	1.47	62	1.59	0.81	20	2.94	3	1.40	0.18
Neonatal Ward	104	15.09	284	7.34	< 0.05	55	8.34	18	7.06	0.52

## Discussion

### Main findings

Vaginal birth, as a preferred delivery method, has many benefits such as less anesthetic and surgical complications, compared to cesarean delivery. While vaginal birth with malpresentation or fetal macrosomia, may cause serious perineal injury which may affect the quality of life. Episiotomy was designed to decrease the rate of serious perineal tears in vaginal deliveries, but routine episiotomy may be unnecessary [25]. For women where a spontaneous vagina birth was anticipated, a policy of selective episiotomy may result in 30% fewer women experiencing severe perineal/vaginal trauma in western country [26–28]. There is a lack of consensus on whether selective or routine episiotomy is preferred in China.

There have been multiple researches comparing selective versus routine episiotomy, they came to conflicting conclusion. Most trials reported no significant difference in the main outcome ‘severe perineal/vaginal trauma’ between the two groups, while some studies suggest a trend toward a greater risk of perineal lacerations in selective episiotomy [26, 29], but a randomized controlled clinical trial by Zuleta found that selective episiotomy reduced the rate of severe perineal lacerations [30]. The effect size was different of in each study, probably owing the differences in the definition and the reference group.

Our large population-based study in Shanghai found that mothers with selective episiotomy had no increased risk of severe perineal/vaginal trauma. The incidence of severe perineal lacerations (selective episiotomy 0.04%, routine episiotomy 0.23%) was similar to western developed countries [26]. Better yet, the mothers with selective episiotomy had a lower risk of postpartum hemorrhage, and newborns in the selective episiotomy group had a lower risk of shoulder dystocia and neonatal ward compared to those with routine episiotomy. Our results did not support the view that routine episiotomy was preferred in Shanghai. This observation is in agreement with several randomized controlled studies in western developed countries [26–28].

We observed that patients in tertiary and secondary hospitals with selective episiotomy were at a similar risk of severe perineal lacerations. In tertiary hospitals, newborns in the selective episiotomy group had lower risk of neonatal ward, but in secondary hospitals, newborns in the selective episiotomy group had higher risk of neonatal ward. This may be due to more experienced physicians and more advanced medical facilities in tertiary hospitals. Moreover, compared to patients with routine episiotomy, there was a lower risk of birth injuries, postpartum hemorrhage and shoulder dystocia with selective episiotomy in tertiary care hospitals where obstetricians could be more experienced in fetal assessment/monitoring might have reduced the risk of inappropriate vaginal delivery [31].

There was no difference in risks of severe perineal lacerations between selective episiotomy policy and routine episiotomy policy in gynecology specialty hospitals compared to general hospitals. But in general hospitals, patients with selective episiotomy policy had higher risk of birth injury. In China, patients are more willing to deliver in obstetric hospitals, so doctors in obstetric hospitals have more experience in handling complicated obstetric patients. We believe that obstetric care providers need more experience in the use of selective episiotomy. The injudicious use of selective episiotomy may decrease the rate of episiotomy but increase the risk of significant perineal lacerations. With more standardized training, patients in all hospitals can be handled appropriately under a policy of selective use of episiotomy.

### Strengths

The strengths of this study include a large sample size with the contemporary data on Chinese pregnant women, the use of uniform definitions of study variables collected across all hospitals.

### Limitations

There are some limitations in our study. First, we only investigated short-term neonatal outcomes. We have no data on potential long-term maternal and child health outcomes. Second, our conclusions may be applicable to secondary and tertiary care settings in urban areas only in China. More studies are required to understand whether the findings are applicable to other settings in China.

## Conclusion

Selective episiotomy is safe and has a similar risk of severe perineal/vaginal trauma compared to routine episiotomy in all types of hospitals in Shanghai, China. Selective episiotomy may be advocated in obstetric and tertiary care settings in urban areas in China.

## Supplementary Information

**Additional file 1.**

## Data Availability

The datasets used and/or analysed during the current study are available from the corresponding author on reasonable request.
