# Supplementary material for: Selective versus routine use of episiotomy for vaginal births in Shanghai hospitals, China: a comparison of policies
Source: BMC Pregnancy Childbirth. 2022 Jun 11;22:475. doi: 10.1186/s12884-022-04790-0 (PMC9188710; doi:10.1186/s12884-022-04790-0)
Supplement: Supplementary file 1 — Additional file 1. [file 12884_2022_4790_MOESM1_ESM.docx]

**Table S1.** Characteristics at baseline (unweighted)

| Characteristics | Routine episiotomy (2183) | | Selective episiotomy  (3295) | | P |
| --- | --- | --- | --- | --- | --- |
|  | mean | 95%CI | mean | 95%CI |  |
| age | 27.10 | 26.95-27.26 | 27.94 | 27.83-28.05 | <0.01 |
| ＜20 | 79.00 | 3.32 | 38.00 | 1.15 |  |
| 20-35 | 2063 | 94.50 | 3191 | 96.84 |  |
| 35- | 41 | 1.88 | 66 | 2.00 |  |
| BMI | 21.38 | 21.26-21.50 | 21.21 | 21.12-21.31 | 0.03 |
| ＜18.5 | 297 | 13.61 | 419 | 12.72 |  |
| 18.5-24 | 1520 | 69.63 | 2437 | 73.96 |  |
| 24- | 366 | 16.77 | 439 | 13.32 |  |
|  | n | % | n | % |  |
| Diabetes | 5 | 0.23 | 6 | 0.18 | 0.70 |
| Pre-existing cardiac diseases | 12 | 0.55 | 36 | 1.09 | 0.03 |
| Pre-existing renal disease | 6 | 0.27 | 7 | 0.21 | 0.64 |
| Pre-existing autoimmune disease | 2 | 0.09 | 3 | 0.09 | 0.99 |
| Pre-existing hyperthyroidism | 10 | 0.46 | 18 | 0.55 | 0.65 |
| Pre-existing hypothyroidism | 27 | 1.24 | 36 | 1.09 | 0.62 |

BMI body mass index

| Characteristics | Routine episiotomy (2183) | | Selective episiotomy  (3295) | | P |
| --- | --- | --- | --- | --- | --- |
|  | n | % | n | % |  |
| ART | 25 | 1.15 | 52 | 1.58 | 0.18 |
| Hyperthyroidism | 11 | 0.5 | 19 | 0.58 | 0.72 |
| Hypothyroidism | 190 | 8.7 | 183 | 5.55 | <0.01 |
| Other thyroidism | 22 | 1.01 | 45 | 1.37 | 0.24 |
| PIH | 313 | 14.34 | 400 | 12.14 | 0.02 |
| GDM | 251 | 11.5 | 333 | 10.11 | 0.10 |
| placental abruption | 1 | 0.05 | 5 | 0.15 | 0.25 |
| PROM | 457 | 20.93 | 752 | 22.82 | 0.10 |
| Prenatal stillbirth | 1 | 0.05 | 3 | 0.09 | 0.54 |

**Table S2.** Current pregnancy complications (unweighted)

ART assisted reproductive technology, PIH pregnancy-induced hypertension syndrome，GDM Gestational Diabetes Mellitus，PROM Premature rupture of membranes

**Table S3.** Selective episiotomy versus Routine episiotomy: maternal and neonatal outcomes (unweighted)

| Characteristics | Routine episiotomy  (2183) | | | Selective episiotomy  (3295) | | | | P | |
| --- | --- | --- | --- | --- | --- | --- | --- | --- | --- |
|  | n | | % | n | | % | |  | |
| Severe perineal laceration | 4 | | 0.18 | 2 | | 0.06 | | 0.18 | |
| Induction | 569 | | 26.07 | 1170 | | 35.51 | | <0.01 | |
| Term labor | 394 | | 18.05 | 729 | | 22.12 | | 0.00 | |
| Meconium | 137 | | 6.28 | 164 | | 4.98 | | 0.04 | |
| Placenta accreta | 34 | | 1.56 | 78 | | 2.37 | | 0.04 | |
| Fetal distress | 78 | | 3.57 | 143 | | 4.34 | | 0.16 | |
| Shoulder dystocia | 10 | | 0.46 | 3 | | 0.09 | | 0.01 | |
| Incompletely uterine rupture | 2 | | 0.09 | 4 | | 0.12 | | 0.74 | |
| Episiotomy | 1916 | | 87.77 | 1247 | | 37.85 | | <0.01 | |
| Postpartum hemorrhage | 78 | | 3.57 | 60 | | 1.82 | | <0.01 | |
| Transfusion | 9 | | 0.41 | 14 | | 0.42 | | 0.94 | |
| Puerperal infection | 4 | | 0.18 | 13 | | 0.39 | | 0.17 | |
| Vacuum or forceps delivery | | 107 | 4.90 | | 145 | | 4.41 | | 0.26 |
| Apgar≤7 (5min) | 4 | | 0.18 | 11 | | 0.33 | | 0.30 | |
| Birth injury | 450 | | 20.61 | 908 | | 27.56 | | <0.01 | |
| Congenital malformation | 41 | | 1.88 | 44 | | 1.34 | | 0.11 | |
| Neonatal Ward | 172 | | 7.88 | 225 | | 6.83 | | 0.14 | |
|  | mean | | 95%CI | mean | | 95%CI | |  | |
| Birthweight | 3278.8 | | 3263.3-3294.3 | 3290.7 | | 3277.9-3303.4 | | 0.25 | |

**Table S4.** Selective episiotomy versus Routine episiotomy: risk of maternal and neonatal outcomes (unweighted)

| Characteristics | Rude OR | 95%CI | P | Adjusted OR | 95%CI | P |
| --- | --- | --- | --- | --- | --- | --- |
| Meconium | 0.782 | 0.619- 0.988 | 0.04 | 0.808 | 0.622-1.050 | 0.11 |
| Fetal distress | 1.224 | 0.924-1.622 | 0.16 | 1.573 | 1.157- 2.138 | <0.01 |
| Shoulder dystocia | 0.198 | 0.054-0.72 | 0.01 | 0.192 | 0.042- 0.873 | 0.03 |
| Incompletely uterine rupture | 1.325 | 0.243-7.241 | 0.75 | 1.587 | 0.254-9.905 | 0.62 |
| Episiotomy | 0.085 | 0.073- 0.098 | <0.01 | 0.103 | 0.089-0.121 | <0.01 |
| Postpartum hemorrhage | 0.501 | 0.356-0.704 | <0.01 | 0.476 | 0.323-0.701 | <0.01 |
| Severe perineal laceration | 0.331 | 0.061-1.808 | 0.20 | 0.433 | 0.066-2.831 | 0.38 |
| Transfusion | 1.031 | 0.445-2.385 | 0.94 | 0.815 | 0.309-2.149 | 0.68 |
| Puerperal infection | 2.158 | 0.703-6.626 | 0.18 | 2.256 | 0.668-7.614 | 0.19 |
| Apgar 5min | 1.825 | 0.580-5.738 | 0.30 | 2.232 | 0.663-7.506 | 0.19 |
| Birth injury | 1.465 | 1.288-1.666 | <0.01 | 1.228 | 1.065-1.417 | <0.01 |
| Neonatal Ward | 0.857 | 0.697-1.053 | 0.14 | 1.098 | 0.874-1.380 | 0.42 |

The ORs were adjusted for maternal age at childbirth, BMI, the use of assisted reproductive technology, induced labor, pregnancy-induced hypertension, preexisting diabetes, hypothyroidism, pre-existing cardiac diseases and Placenta accrete.

**Table S5.** Selective episiotomy versus Routine episiotomy in Secondary hospitals and Tertiary hospitals: maternal and neonatal outcomes (unweighted)

|  | Secondary hospitals （2681） | | | | | Tertiary hospitals（2797） | | | | |
| --- | --- | --- | --- | --- | --- | --- | --- | --- | --- | --- |
| Characteristics | Routine episiotomy （861） | | Selective episiotomy （1820） | | P | Routine episiotomy （1322） | | Routine episiotomy （ 1475） | | P |
|  | n | % | n | % |  | n | % | n | % |  |
| Severe perineal laceration | 0 | 0 | 1 | 0.05 | 0.49 | 4 | 0.30 | 1 | 0.07 | 0.14 |
| Term labor | 202 | 23.46 | 567 | 31.15 | <0.01 | 367 | 27.76 | 603 | 40.88 | <0.01 |
| Doula | 332 | 38.56 | 1216 | 66.81 | <0.01 | 92 | 6.96 | 785 | 53.22 | <0.01 |
| Meconium | 43 | 4.99 | 43 | 2.36 | <0.01 | 94 | 7.11 | 121 | 8.20 | 0.28 |
| Placenta accreta | 9 | 1.05 | 47 | 2.58 | <0.01 | 25 | 1.89 | 31 | 2.10 | 0.69 |
| Fetal distress | 11 | 1.28 | 68 | 3.74 | <0.01 | 67 | 5.07 | 75 | 5.08 | 0.98 |
| Shoulder dystocia | 0 | 0 | 1 | 0.05 | 0.49 | 10 | 0.76 | 2 | 0.14 | 0.01 |
| Incompletely uterine rupture | 1 | 0.12 | 2 | 0.11 | 0.9639 | 1 | 0.08 | 2 | 0.14 | 0.63 |
| Episiotomy | 803 | 93.26 | 656 | 36.04 | <0.01 | 1113 | 84.19 | 591 | 40.07 | <0.01 |
| Postpartum hemorrhage | 27 | 3.14 | 29 | 1.59 | <0.01 | 51 | 3.86 | 31 | 2.10 | <0.01 |
| Severe perineal laceration | 0 | 0 | 1 | 0.05 | 0.49 | 4 | 0.30 | 1 | 0.07 | 0.14 |
| Transfusion | 1 | 0.12 | 5 | 0.27 | 0.42 | 8 | 0.61 | 9 | 0.61 | 0.99 |
| Puerperal infection | 2 | 0.23 | 8 | 0.44 | 0.41 | 2 | 0.15 | 5 | 0.34 | 0.32 |
| Apgar≤7 (5min) | 0 | 0 | 5 | 0.27 | 0.12 | 4 | 0.30 | 6 | 0.41 | 0.64 |
| Birth injury | 187 | 21.72 | 611 | 33.57 | <0.01 | 263 | 19.89 | 297 | 20.14 | 0.87 |
| Congenital malformation | 5 | 0.58 | 19 | 1.04 | 0.23 | 36 | 2.72 | 25 | 1.69 | 0.06 |
| Neonatal Ward | 16 | 1.86 | 116 | 6.37 | <0.01 | 156 | 11.80 | 109 | 7.39 | <0.01 |
|  | mean | 95%CI | mean | 95%CI |  | mean | 95%CI | mean | 95%CI |  |
| Birthweight | 3276.1 | 3253.0-3299.2 | 3296.1 | 3279.0-3313.1 | 0.18 | 3280.5 | 3259.8-3301.3 | 3284.0 | 3264.9-3303.2 | 0.81 |

**Table S6.** Selective episiotomy versus Routine episiotomy in Secondary hospitals and Tertiary hospitals: risk of maternal and neonatal outcomes (unweighted)

| Characteristics | Secondary hospitals （2681） | | | | | | Tertiary hospitals（2797） | | | | | |
| --- | --- | --- | --- | --- | --- | --- | --- | --- | --- | --- | --- | --- |
|  | Rude OR | 95%CI | P | Adjusted OR | 95%CI | P | Rude OR | 95%CI | P | Adjusted OR | 95%CI | p |
| Severe perineal laceration | >999 | <0.01->99 | 0.9529 | 334.624 | <0.01 ->99 | 0.9380 | 0.224 | 0.025-2.003 | 0.18 | 0.368 | 0.038-3.542 | 0.39 |
| Meconium | 0.460 | 0.299-0.708 | <0.01 | 0.531 | 0.333-0.847 | <0.01 | 1.167 | 0.882-1.545 | 0.28 | 0.995 | 0.710-1.394 | 0.98 |
| Fetal distress | 2.998 | 1.577-5.697 | <0.01 | 3.855 | 1.981-7.504 | <0.01 | 1.003 | 0.716-1.407 | 0.98 | 1.139 | 0.770-1.686 | 0.51 |
| Shoulder dystocia | >999 | <0.001->999 | 0.9529 | >999 | <0.001->999 | 0.9261 | 0.178 | 0.039-0.815 | 0.03 | 0.163 | 0.025-1.058 | 0.06 |
| Episiotomy | 0.041 | 0.031-0.054 | <0.01 | 0.040 | 0.030-0.054 | <0.01 | 0.126 | 0.105-0.150 | <0.01 | 0.157 | 0.128-0.193 | <0.01 |
| Postpartum hemorrhage | 0.500 | 0.294-0.850 | 0.01 | 0.476 | 0.266-0.852 | 0.01 | 0.535 | 0.340-0.841 | <0.01 | 0.441 | 0.251-0.773 | 0.01 |
| Transfusion | 2.369 | 0.276-20.306 | 0.4314 | 3.026 | 0.306-29.897 | 0.3433 | 1.008 | 0.388-2.621 | 0.99 | 0.740 | 0.214-2.557 | 0.63 |
| Birth injury | 1.822 | 1.508- 2.200 | <0.01 | 1.554 | 1.266-1.906 | <0.01 | 1.015 | 0.843-1.222 | 0.87 | 0.887 | 0.711-1.106 | 0.29 |
| Neonatal Ward | 3.595 | 2.118- 6.103 | <0.01 | 4.166 | 2.408- 7.206 | <0.01 | 0.596 | 0.461-0.771 | <0.01 | 0.767 | 0.568-1.034 | 0.08 |

The ORs were adjusted for maternal age at childbirth, BMI, birthweight, the use of assisted reproductive technology, induced labor, pregnancy-induced hypertension, preexisting diabetes, hypothyroidism, pre-existing cardiac diseases and Placenta accrete.

|  | Obstetric hospitals （3238） | | | | | General hospitals (2240) | | | | |
| --- | --- | --- | --- | --- | --- | --- | --- | --- | --- | --- |
| Characteristics | Routine episiotomy （629） | | Selective episiotomy （2609） | |  | Routine episiotomy （1554） | | Selective episiotomy （686） | |  |
|  | n | % | n | % | P | n | % | n | % | P |
| Severe perineal laceration | 21 | 3.34 | 40 | 1.53 | <0.01 | 57 | 3.67 | 20 | 2.92 | 0.37 |
| Term labor | 174 | 27.66 | 883 | 33.84 | <0.01 | 395 | 25.42 | 287 | 41.84 | <0.01 |
| Doula | 71 | 11.29 | 1757 | 67.34 | <0.01 | 353 | 22.72 | 244 | 35.37 | <0.01 |
| Meconium | 46 | 7.31 | 139 | 5.33 | 0.05 | 91 | 5.86 | 25 | 3.64 | 0.03 |
| Placenta accreta | 12 | 1.91 | 65 | 2.49 | 0.39 | 22 | 1.42 | 13 | 1.9 | 0.40 |
| Fetal distress | 47 | 7.47 | 112 | 4.29 | <0.01 | 31 | 1.99 | 31 | 4.52 | <0.01 |
| Shoulder dystocia | 9 | 1.43 | 3 | 0.11 | <0.01 | 1 | 0.06 | 0 | 0 | 0.51 |
| Incompletely uterine rupture | 0 | 0 | 4 | 0.15 | 0.33 | 2 | 0.13 | 0 | 0 | 0.3472 |
| Postpartum hemorrhage | 475 | 75.52 | 833 | 31.93 | <0.01 | 1441 | 92.73 | 414 | 60.35 | <0.01 |
| Transfusion | 1 | 0.16 | 1 | 0.04 | 0.27 | 3 | 0.19 | 1 | 0.15 | 0.81 |
| Puerperal infection | 2 | 0.32 | 8 | 0.31 | 0.96 | 7 | 0.45 | 6 | 0.87 | 0.22 |
| Postpartum hemorrhage | 2 | 0.32 | 11 | 0.42 | 0.71 | 2 | 0.19 | 2 | 0.46 | 0.40 |
| Apgar≤7 (5min) | 0 | 0 | 8 | 0.31 | 0.16 | 4 | 0.26 | 3 | 0.44 | 0.48 |
| Birth injury | 61 | 9.7 | 617 | 23.65 | <0.01 | 389 | 25.03 | 291 | 42.42 | <0.01 |
| Congenital malformation | 7 | 1.11 | 35 | 1.34 | 0.65 | 34 | 2.19 | 9 | 1.31 | 0.16 |
| Neonatal Ward | 72 | 11.45 | 179 | 6.86 | <0.01 | 100 | 6.44 | 46 | 6.71 | 0.81 |
|  | mean | 95%CI | mean | 95%CI |  | mean | 95%CI | mean | 95%CI |  |
| Birthweight | 3279.5 | 3250.3-3308.6 | 3297.3 | 3283.0-3311.5 | 0.28 | 3278.5 | 3260.1-3296.9 | 3265.6 | 3237.1-3294 | 0.45 |

**Table S7.** Selective episiotomy versus Routine episiotomy in Obstetric hospitals and General hospitals: maternal and neonatal outcomes (unweighted)

**Table S8.** Selective episiotomy versus Routine episiotomy in Obstetric hospitals and General hospitals: risk of maternal and neonatal outcomes (unweighted)

The ORs were adjusted for maternal age at childbirth, BMI, birthweight, the use of assisted reproductive technology, induced labor, pregnancy-induced hypertension, preexisting diabetes, hypothyroidism, pre-existing cardiac diseases and Placenta accrete.

| Characteristics | Obstetric hospitals （3238） | | | | | | General hospitals (2240) | | | | | |
| --- | --- | --- | --- | --- | --- | --- | --- | --- | --- | --- | --- | --- |
|  | Rude OR | 95%CI | P | Adjusted OR | 95%CI | P | Rude OR | 95%CI | P | Adjusted OR | 95%CI | P |
| Meconium | 0.713 | 0.505-1.007 | 0.06 | 0.785 | 0.525-1.173 | 0.24 | 0.608 | 0.387-0.956 | 0.03 | 0.698 | 0.438-1.112 | 0.13 |
| Fetal distress | 0.555 | 0.390-0.790 | <0.01 | 0.842 | 0.565-1.255 | 0.40 | 2.325 | 1.402-3.857 | <0.01 | 2.585 | 1.529-4.371 | <0.01 |
| Episiotomy | 0.152 | 0.125-0.186 | <0.01 | 0.187 | 0.150-0.233 | <0.01 | 0.119 | 0.093-0.153 | <0.01 | 0.124 | 0.096-0.160 | <0.01 |
| Postpartum hemorrhage | 0.451 | 0.264-0.770 | <0.01 | 0.492 | 0.256-0.946 | 0.03 | 0.789 | 0.470-1.323 | 0.37 | 0.716 | 0.416-1.235 | 0.23 |
| Severe perineal laceration | 0.241 | 0.015-3.855 | 0.31 | 0.233 | 0.003-16.814 | 0.50 | 0.755 | 0.078-7.269 | 0.81 | 0.815 | 0.076-8.786 | 0.87 |
| Transfusion | 1.327 | 0.293-6.001 | 0.71 | 0.916 | 0.150-5.616 | 0.92 | 1.950 | 0.653-5.824 | 0.23 | 1.438 | 0.414-5.002 | 0.57 |
| Birth injury | 2.884 | 2.181-3.812 | <0.01 | 2.244 | 1.657-3.040 | <0.01 | 2.207 | 1.825-2.668 | <0.01 | 1.914 | 1.569-2.335 | <0.01 |
| Neonatal Ward | 0.570 | 0.427-0.761 | <0.01 | 0.662 | 0.473-0.926 | 0.02 | 1.045 | 0.728-1.500 | 0.81 | 1.338 | 0.917-1.953 | 0.13 |
